# Lipid regulation in *Brassica napus*: spatiotemporal studies to enhance our understanding

**DOI:** 10.3389/fpls.2026.1813997

**Published:** 2026-04-29

**Authors:** Pan Liao, Tamara Lechon, John L. Harwood, Simon Scofield

**Affiliations:** 1 Department of Biology, Hong Kong Baptist University, Kowloon Tong, Hong Kong SAR, China; 2 State Key Laboratory of Agrobiotechnology (CUHK), Shatin, Hong Kong SAR, China; 3 School of Biosciences, Cardiff University, Cardiff, United Kingdom

**Keywords:** *Brassica napus*, canola, fatty acid, lipid regulation, MALDI- MS, oilseed rape

## Abstract

*Brassica napus* (and related *Brassica* species) is the world’s third most important oil crop, providing around 13% of the total global vegetable oil. There are two basic types of cultivars - those with significant erucate being used for renewable chemicals, while low-erucate varieties (the majority) supply the food industry. *B. napus* oil is particularly enriched in oleate but also contains a nutritionally desirable ratio of *n*-3 to *n*-6 polyunsaturated fatty acids. In this review we note the overall importance of rapeseed oil and describe in detail how it is biosynthesised, mainly through the classic Kennedy pathway with additional reactions. We then discuss very recent advances in our understanding of how biosynthesis is regulated and spatiotemporal aspects of oil accumulation in the crop. Both biotic and abiotic environmental effects on *B.napus* yields are then summarized. Recently, MALDI-MSI has been developed for lipids and its ability to reveal spatial and temporal differences in lipid species distribution has proven especially useful. The technique has exposed unsuspected details in metabolism as well as confirming other reported aspects of lipid biochemistry. The similar, but not identical, lipid metabolism in Arabidopsis has facilitated many of the advances in *B. napus* and it is anticipated that the momentum of new discoveries will continue to be rapid and significant.

## 1 Introduction

Brassica oilseed species were one of the first plants domesticated by humans. They were cultivated in India 3000–4000 years ago and in China 2000–2500 years ago (McVetty et al., 2016). In the Middle Ages cultivation extended to Europe and rapeseed oil was used in the Rhineland as a cooking fat and lamp oil (Gunstone, 2004). Of the 37 species in the Brassica genus, the four most widely cultivated are *B. napus*, *B. juncea*, *B. rapa* and *B. carinata*. They are also notable as being one of the few oilseed species which can be grown in temperate climates (Gunstone and Harwood, 2007).

Rapeseed is currently the third most important oil crop (after oil palm and soybean) and is a major oil crop in Canada, China, Europe and India (Harwood et al., 2017). *B. rapa* is the most cold-hardy and is important in Western Canada, while ecotypes of this species are grown in the Indian subcontinent. *B. napus* is the main rapeseed species grown in Europe, Canada and China. Spring and winter-sown varieties are available with the latter producing higher yields under appropriate growing conditions. *B. juncea* is important in Northern India and parts of China where its tolerance of drier conditions is useful, while *B. carinata* is cultivated in Ethiopia and nearby African countries (Gunstone and Harwood, 2007). A fifth species, *B. campestris* has been used recently as a spring crop in Scandinavia, North West China and some parts of Canada.

*B. carinata, B. juncea* and *B. napus* are amphidiploids combining chromosome sets of the diploid parents *B. nigra, B. juncea* and *B. rapa* (McVetty et al., 2016). *B. napus* is an allotetraploid crop resulting from the interspecies breeding of varieties of *B. oleracea* and *B. rapa* (Chalhoub et al., 2014; An et al., 2019). This makes elucidating some of the regulatory molecular mechanisms relating to lipid biochemistry more complicated, and in some cases, imprecise.

Earlier varieties of rapeseed frequently contained high amounts of erucic acid and substantial quantities of glucosinolates. Some initial studies (e.g. Heijkenskjold and Ernster, 1975) indicated that erucic acid might give rise to myocardial lipidosis and this led to the breeding of ‘canola’, a low-erucic acid *B. napus* line. Canola also had reduced levels of glucosinolates, which caused low nutritional properties and limited palatability for livestock (Daun et al., 2011). More recently, there have been debates about how toxic erucic acid may be (Weselake et al., 2017; Galanty et al., 2023; Wang et al., 2024). Indeed, rapeseed lines containing substantial amounts of erucic acid are consumed regularly in China and India.

Currently there are two main types of *B. napus*, a high erucic acid type (HEAR) which is used industrially as a lubricant, and a low erucic acid type (LEAR) which is used nutritionally (see **Table 1**). The latter is usually called ‘canola’ in North America. As described in detail by McVetty et al. (2016), canola breeding objectives included not only seed quality but also seed yield, environmental adaptation, disease and pest resistance and herbicide tolerance.

**Table 1:** Fatty acid composition of typical low or high erucate *B. napus* oil.

Fatty acid	LEAR (%)		HEAR (%)
Palmitic	3.9	4.0
Stearic	1.6	1.0
Oleic	60.7	14.2
Linoleic	20.6	13.5
α-Linolenic	9.9	9.3
Arachidic	0.6	0.8
Docosanoic	0.4	0.8
Docosenoic	1.1	9.3
Erucic	0.3	46.6

The compositions are based on the average compositions for different LEAR and HEAR *B. napus* lines. Note that the % compositions will vary from year to year due to different growing conditions. For more details and examples see Gunstone and Harwood (2007).

Growth of rapeseeds and, hence, production of rapeseed oils varies somewhat from year to year, depending on environmental factors as well as political considerations. The figures reported usually do not distinguish between LEAR and HEAR varieties, although the former types represent up to 90% of the total. Of the main counties/regions, Canada is a major exporter, China is an important grower and importer while India and Northern Europe are major growers and producers of oil (Gunstone and Harwood, 2007; **Table 2**).

**Table 2:** Production of rapeseed by country since 2000.

Country/region	2000	2012	2024
Canada	7.2	15.4	19.2
EU	11.3	19.2	16.9
China	11.3	14.0	15.8
India	5.8	6.8	11.5

The figures are based on average values in the literature and online and are in millions of tonnes. The total production of rapeseed in these four countries/regions represents about 74% of the total world output in 2004. Figures for rapeseed oil production show that 83% of the world output was accounted for by these four regions in 2005 (Gunstone and Harwood, 2007).

Over the years many different cultivars have been developed (Gunstone and Harwood, 2007; McVetty et al., 2016). Many of these were aimed at particular food/feed markets but, in addition, super high erucic acid rapeseed (SHEAR) with up to 60% erucic acid has been developed for chemical applications (see Scarth and Tang, 2006). HEAR varieties of rapeseed have industrial uses as detergents, cosmetics, plastic additives and as an ingredient in high-temperature lubricants. The fatty acid composition of typical LEAR and HEAR rapeseed oils are shown in **Table 1**. HEAR oils contrast with LEAR oils in that the high oleate content of the latter has been elongated to eicosenoic (~10%) and erucic (~45%) acids while desaturation to linoleic acid is also significantly reduced.

Typical LEAR varieties of *B. napus* provide oils with a very high oleic acid proportion (over 60%). They are low in saturated fatty acids (**Table 1**) and contain around 30% of the dietary indispensable polyunsaturated fatty acids (PUFAs), linoleic and alpha-linolenic. While both of these are needed for good health (Spector and Kim, 2015), there has been increasing awareness that their dietary ratio is important because they give rise to lipid mediators which have markedly different physiological and biochemical effects (Christie and Harwood, 2020; Calder, 2020). LEAR (and canola) oils are notable in having a ratio of n-6:n-3 PUFAs of around 2:1 which is considered to be helpful for good human health (Harwood, 2019, Harwood, 2023). In particular, there has been much attention to the role of n-3 PUFAs in their conversion to pro-resolving mediators and the latter’s potential role in health (Chiang and Serhan, 2020; Harwood, 2023).

As for other crops, triacylglycerol (TAG) is the main accumulating lipid and represents over 95% of the total lipids in *B*. *napus* seeds (Perry and Harwood, 1993a). TAG is produced by the four steps of the Kennedy pathway (Kennedy, 1957; Kennedy, 1961) and associated reactions, as described in section 2. The availability of different acyl-CoA substrates together with the substrate selectivities of the three acyltransferases of the Kennedy pathway gives rise to different molecular species of TAG in the accumulating oil. In typical LEAR oils the major TAG species are OOO (22%), LOO (22%), LnOO (10%), LLO (9%), LnLO (8%), LOP (6%) and POO (5%), where L, Ln, O and P refer to linoleic, linolenic, oleic and palmitic acids, respectively (Gunstone and Harwood, 2007). For more detail about the distribution of different molecular species of TAG in various rapeseed species see (Gunstone et al., 2007). Within the glycerol backbone, the PUFA components are concentrated at the *sn*-2 position, as they are in most other oil crops (Gunstone et al., 2007). While there are continual efforts to increase oil yields (McVetty et al., 2016; Weselake et al., 2024), typical lipid contents for LEAR seeds are around 45% dry weight. Seed yields are given as an average of 1.73 t/hectare (with the European Union at 3.40, China at 1.78, Canada at 1.58 and India at 0.91) (Gunstone and Harwood, 2007). However, these figures do not distinguish between the different species grown in these four regions.

There have been and currently are numerous breeding programmes to improve the quantity and quality of rapeseed oil (McVetty et al., 2016). Furthermore, rapeseed is relatively easy to transform. The *B. napus* genome was sequenced in 2014 (Chalhoub et al., 2014) and this has helped efforts to develop the crop both through conventional breeding and genetic modification. Genetically engineered lines are grown extensively in North America and their production and utilisation have been described (Weselake et al., 2017). In addition, work to increase oil yields in *B. napus* and other rapeseeds has been reviewed (Weselake et al., 2024).

## 2 Lipid biosynthesis

### 2.1 *De novo* formation of fatty acids

For a background summary of earlier work, please refer to Harwood (2005). *De novo* biosynthesis of fatty acids clearly needs a source(s) of carbon. *Arabidopsis thaliana* (Arabidopsis) has often been used as a model system for Brassica spp., although it should be stressed that, although often similar, lipid biochemistry in Arabidopsis is not identical to *B. napus* in all respects (Li et al., 2006). Work in the 1980s and 1990s suggested several sources of carbon for fatty acid biosynthesis in Arabidopsis but it was agreed generally that plastid pyruvate dehydrogenase was the major source of acetyl-CoA for fatty acid formation (Harwood, 2005). At early stages of seed development glucose-6-phosphate can enter oilseed rape plastids via a specific transporter and is a better source than exogenous pyruvate. However, at later stages when oil accumulation is rapid, glucose-6-phosphate is a substrate for the pentose phosphate pathway and pyruvate is preferred for fatty acid production (Eastmond and Rawsthorne, 2000). Indeed, the pyruvate transporter has been shown to be important for oil production in *B. napus* (Tang et al., 2022a). A useful review including aspects of carbon supply under various conditions has been published (Rawsthorne, 2002). Two recent papers highlight the relevance of phosphoenolpyruvate (Tang et al., 2022b) and ATP transport into plastids (Hong et al., 2022?) for maintaining seed oil accumulation in *B. napus*.

The overall pathway for *de novo* fatty acid formation involves the reactions of acetyl-CoA carboxylase (ACCase) and a Type II fatty acid synthase in plants (Harwood, 2005). Acetyl-CoA carboxylase catalyses the first committed step in fatty acid production and, hence, acyl lipid formation. It is a biotin-containing enzyme which uses ATP to catalyse the formation of malonyl-CoA from bicarbonate and acetyl-CoA. There are two forms of ACCase in plants, one in the plastid (for *de novo* synthesis) and one in the cytosol (for elongation of pre-formed fatty acids). These ACCases have distinct properties (Alban et al., 1994; Herbert et al., 1996) and their structures have been reviewed (Harwood, 2005). Although both isoforms have similar molecular masses, when purified from monocotyledous plants such as maize, the major ACCase (227 KDa), unlike the minor cytosolic enzyme (219 KDa) was poorly sensitive to inhibition by graminicide herbicides (Herbert et al., 1996). Moreover, they could be distinguished easily using antibodies (Egli et al., 1993) and had distinct sensitivities to acyl-CoAs (see Harwood, 2005). The sensitivity of the multifunctional form of ACCase in monocotyledons to herbicides was utilized in barley and maize to determine flux control coefficients for lipid synthesis in the light. Data showed that ACCase controlled 45-61% of the total carbon regulation (Page et al., 1994).

In contrast to the Poaceae, plants such as *Brassica* spp. have a plastid ACCase which contains subunits (Harwood, 2005). Since it is such an important enzyme for fatty acid biosynthesis, it is not surprising that ACCase is subject to acute and chronic regulation. Early experiments in this area have been discussed in detail (Harwood, 1996; Ohlrogge and Jaworski, 1997). Reversible phosphorylation associated with redox modulation (Sasaki et al., 1997) or high ATP levels (Savage and Ohlrogge, 1999) were implicated (Harwood, 2005). More recently, attention has focused on biotin attachment domain-containing (BADC) proteins (Liu et al., 2019). These were found to interact with the biotin-carboxyl carrier protein (BCCP) subunit of the heteromeric ACCase (Salie et al., 2016). BADC proteins can inhibit ACCase activity markedly (Salie et al., 2016; Keereetaweep et al., 2018) and gene silencing of BADC isoform 1 in Arabidopsis can increase oil accumulation significantly (Salie et al., 2016). In addition, there are carboxyltransferase interactors which can bind to the alpha-carboxyltransferase subunit of heteromeric ACCase. This allows ‘docking’ of ACCase to the plastid envelope in leaves (Ye et al., 2020) and could be of significance in oil seeds also (Weselake et al., 2024).

Once malonyl-CoA is formed by ACCase, the plastid fatty acid synthase (FAS) takes over. The plant FAS is a Type II dissociable multienzyme complex, similar to the *E.coli* system. Of the individual components of the plant FAS, two acyltransferases have been detected. Malonyl-CoA: ACP transferase is critical for generation of the malonyl-ACP units used by all three condensing enzymes. The second, an acetyl-CoA: ACP transacylase, is of doubtful physiological importance (Harwood, 2005).

**Figure 1:**
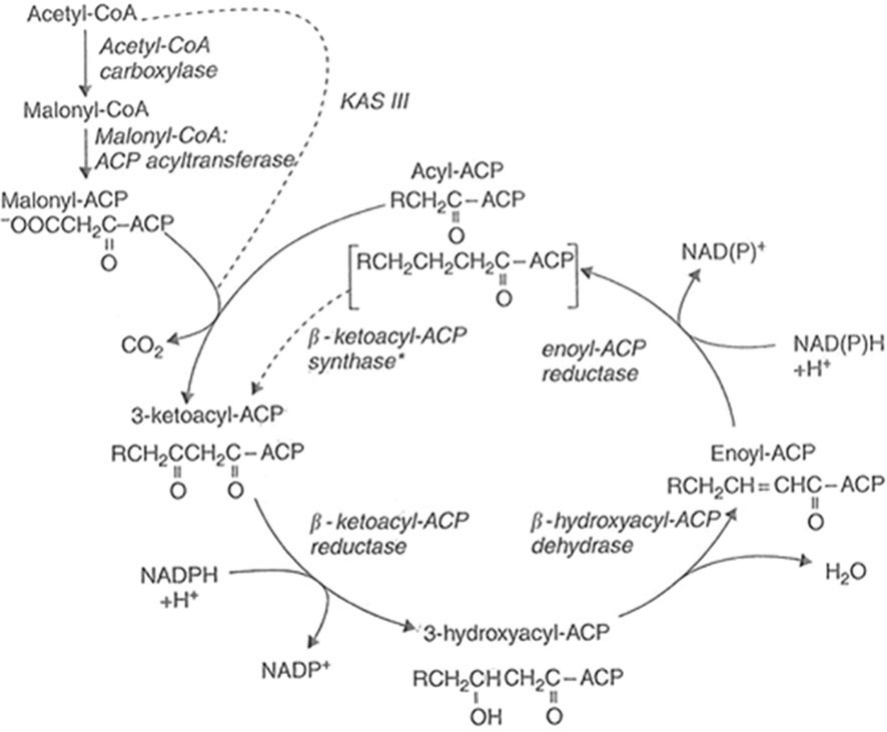
The overall reaction of fatty acid synthase. The first condensation reaction is catalysed by β-ketoacyl-ACP synthase III (KAS III), which uses acetyl-CoA and malonyl-ACP substrates. The next six condensations are catalysed by KAS I while the final reaction between palmitoyl-ACP and malonyl-ACP utilises KAS II. Adapted from Harwood (2005).

The three condensing enzymes are beta-ketoacyl-ACP synthetases (KASs) I, II and III. The first condensation uses KAS III and this was discovered by analogy to the FabH protein in *E. coli* (Jaworski et al., 1989). It was later found to have widespread occurrence in different higher plant species (Walsh et al.,1990: Dehesh et al., 2001) and condenses acetyl-CoA directly with malonyl-ACP, yielding a 4C-keto-intermediate (**Figure 1**). KAS I is used for the formation of 6–16 carbon fatty acids while KAS II was discovered by being arsenite-sensitive (Harwood and Stumpf, 1971) and is specific for production of stearate (Harwood, 2005).

Once condensation has taken place, a cycle of reduction, dehydration and a second reduction takes place to yield fatty acid products containing two carbons extra in each cycle (**Figure 1**). Details of these four reactions, the acyl carrier proteins in plants and associated genes are given in Harwood (2005).

In most plants, palmitate and oleate are the main products of *de novo* synthesis with oleate being formed from stearoyl-ACP by a soluble delta-9-desaturase within the plastid (Shanklin and Cahoon, 1998). The acyl chains are released from their ACP-esters by the action of thioesterases (fatty acid thioesterases, FATs). The latter terminate synthesis by releasing non-esterified (‘free’) fatty acids and also make them available for the so-called ‘eukaryotic pathways’ of complex lipid metabolism outside the plastid (Browse and Somerville, 1991; Harwood, 1996). The acyl-ACP thioesterases were purified from oilseed rape (Hellyer and Slabas, 1990). Once studied, it was clear that there were two types (Jones et al.,1995; Voelker et al., 1997). FATA has high activity with oleoyl-ACP but much less with saturated substrates while FATB has highest activity with saturated substrates but also good activity with oleoyl-ACP and other unsaturated substrates. For further discussion of the FAT enzymes and regulation on *de novo* fatty acid formation see (Harwood, 2005).

### 2.2 Lipid assembly

Non-esterified fatty acids (NEFAs) released by FAT activity have to be re-esterified to CoA. Long-chain acyl-CoA synthetases (LACS) carry out this and, in Arabidopsis, there are 9 isoforms (Zhao et al., 2010). Most of these are involved in production of acyl-CoAs throughout the plant’s life, and genes encoding these enzymes were identified and cloned to allow characterisation of their substrate selectivities. Of the LACS enzymes, LACS1 and LACS9 were highly expressed in developing seeds (Shockey et al., 2002). For *B. napus*, LACS2 has also been suggested to be important for seed oil accumulation (Ding et al., 2020).

The NEFAs released by FAT activity were first proposed to be transported out of the plastid using an ABC transporter (Kim et al., 2013). Since then, characterisation of a fatty acid transporter termed FAX1 (Li et al., 2015) has been shown to be important and further work has shown that this fatty acid transporter 1 enhances seed oil content in *B. napus* (Xiao et al., 2021; Li et al., 2022) as AtFAX1 does in Arabidopsis (Tian et al., 2019).

For *B. napus* HEAR, modification of fatty acids in the endoplasmic reticulum involves elongases and these have been targeted to increase erucic acid percentages (e.g. Katavic et al., 2000). This process uses membrane-bound substrates and NADH as the main reducing coenzyme. Elongation takes place in four steps, analogous to fatty acid synthase (Cassagne et al., 1994). The first reaction is catalysed by condensing enzymes (3-ketoacyl-CoA synthase) and it is this reaction that prescribes the chain length for elongation (Miller and Kunst, 1997). Some details of the elongation reactions in plants have been reviewed (Hildebrand et al., 2005; Kunst et al., 2005) with more specific details in (Trenkamp et al., 2004; Haslam and Kunst, 2013). More recent updates are (Xue et al., 2020; Batsale et al., 2021, Batsale et al., 2023). There are 21 condensing enzymes in Arabidopsis (Batsale et al., 2023) and it was these that were targeted when breeding LEAR varieties of *B. napus* (see section 1). In general, elongation of long-chain fatty acids plays a pivotal role for the production of surface coverings, waxes, cutin and suberin (Kunst et al., 2005).

For triacylglycerol (TAG) and most other acyl lipids, the glycerol backbone is provided by *sn*-3 glycerol-3-phosphate (G3P) which is produced from the glycolytic intermediate dihydroxyacetone phosphate by *sn*-3 glycerol 3-phosphate dehydrogenase (Vigeolus et al., 2007).

**Figure 2:**
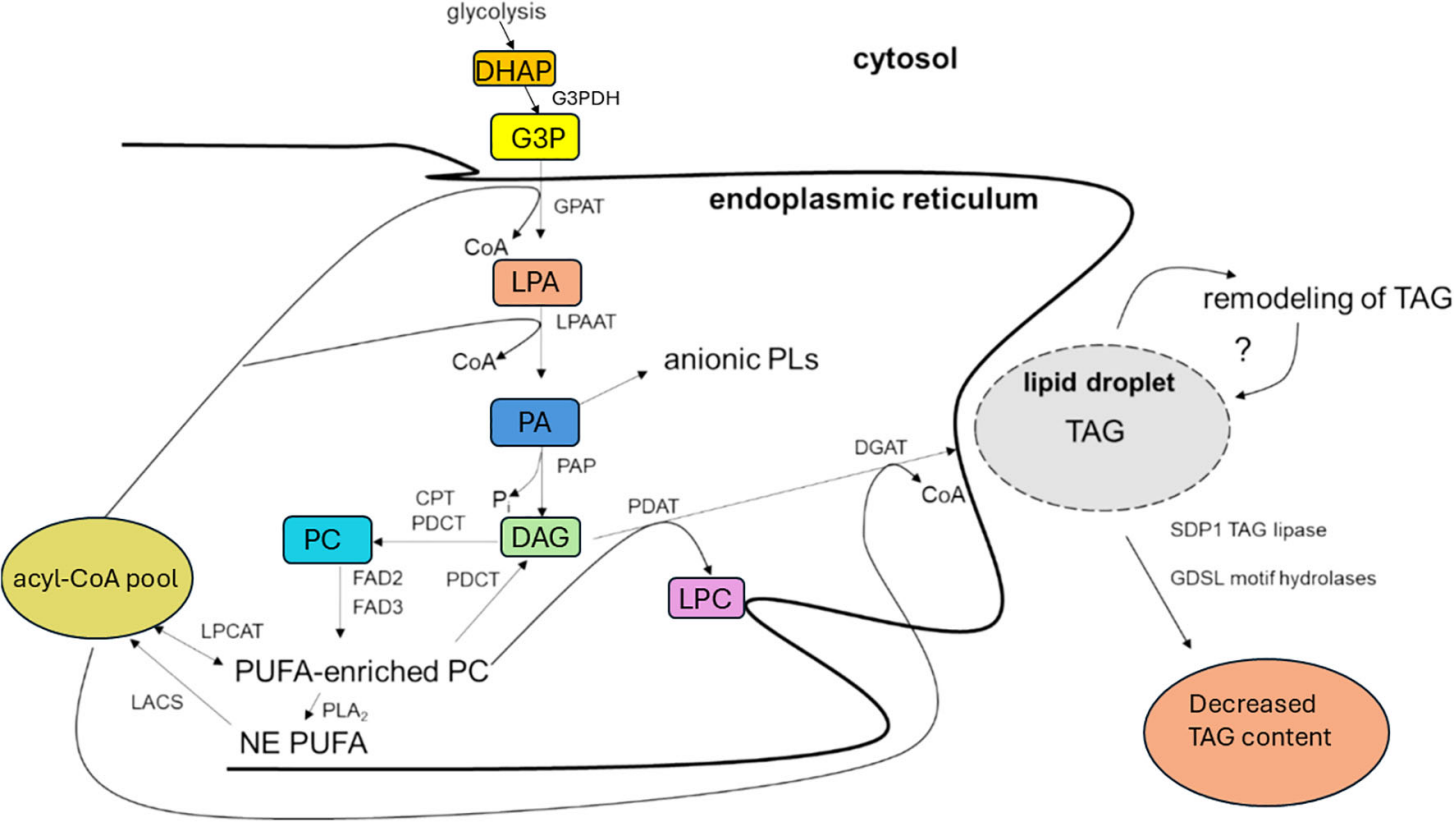
Generalized overview of the triacylglycerol (TAG) biosynthesis in developing seeds of oleaginous plants producing TAG containing polyunsaturated fatty acids (PUFAs). The Kennedy pathway is shown in relation to some possible acyl-trafficking reactions along with phospholipid:diacylglycerol acyltransferase (PDAT) action. Possible specialized pools of 1, 2-diacyl-*sn*-glycerol (DAG), including DAG synthesized *de novo* in the Kennedy pathway and PC-modified DAG are not specifically depicted. Phosphatidylethanolamine can also serve as an acyl donor for the PDAT-catalysed reaction. Other abbreviations: CPT, CDP-choline:1,2-diacyl-*sn*-glycerol cholinephosphotransferase; FA, fatty acid; FAD, fatty acid desaturase; GPAT, acyl-CoA:*sn*-glycerol-3-phosphate acyltransferase; G3P, *sn*-glycerol-3-phosphate; LACS, long-chain acyl-CoA synthetase; LPA, lysophosphatidic acid; LPAAT, acyl-CoA:lysophosphatidic acid acyltansferase; LPC, lysophosphatidylcholine; LPCAT, acyl-CoA:lysophosphatidylcholine acyltransferase; NE, non-esterified; PA, phosphatidic acid; PAP, phosphatidic acid phosphatase; PC, phosphatidylcholine; PDCT, phosphatidylcholine:diacylglycerol; cholinephosphotransferase; Pi, inorganic phosphate; PL, phospholipid; PLA2, phospholipase A2; SDP1, sucrose-dependent 1. Adapted from Weselake et al. (2024).

The overall biosynthetic pathway for TAG production in the endoplasmic reticulum of oilseeds is shown in **Figure 2**. It follows the Kennedy pathway, named after its discoverer (Kennedy, 1957, Kennedy, 1961). To summarise, the pathway involves two sequential acyltransferase steps (glycerol-3-phosphate acyltransferase (GPAT), lysophosphatidic acid acyltransferase (LPAAT), a phosphatase step (phosphatidic acid phosphatase, PAP) and a final diacylglycerol acyltransferase (DGAT) reaction. The overall pathway and its individual enzymes have been well described (Weselake, 2005) and updates provided (Chen et al., 2015; Weselake et al., 2024). The final reaction, catalysed by DGAT, is thought to be a key reaction influencing oil accumulation in rapeseed (Perry and Harwood, 1993a; Perry and Harwood, 1993b; Lung and Weselake, 2006; Weselake et al., 2008, Weselake et al., 2009; Liu et al., 2012; Xu et al., 2018; Chen et al., 2022). DGAT is also considered to have an important influence on the fatty acid proportions in the oil accumulated in *B. napus* (Woodfield et al., 2018).

The Kennedy pathway also gives rise to the synthesis of the major plant membrane acyl lipids, namely phosphoglycerides and glycosylglycerides. Phosphatidic acid (PA) is used as a precursor for anionic phosphoglycerides (inositol lipids, cardiolipin, phosphatidylglycerol) while DAG is used to produce the zwitterionic phosphoglycerides (phosphatidylcholine (PC), phosphatidylethanolamine) and the glycosylglycerides (Dormann, 2005).

As mentioned in Section 1, LEAR varieties of *B. napus* are highly enriched in oleate. Moreover, the polyunsaturated fatty acids linoleic acid and alpha-linolenic acid are found at about 22% and 10%, respectively (Woodfield and Harwood, 2017). These acids are synthesised by fatty acid desaturases (FADs). Although they are formed in plastids by FAD6 and FAD7/8, respectively, for oil formation their main production is by FAD2 and FAD3 on the endoplasmic reticulum (Wallis and Browse, 2002). The main substrate for the FAD2 and FAD3 reactions is the principal phosphoglyceride in the endoplasmic reticulum, phosphatidylcholine, thus emphasising the interactions between different lipids in this organelle (**Figure 2**). Indeed, DAG in the Kennedy pathway can be converted to PC by CDP:1,2-diacyl-*sn*-glycerol cholinephosphotransferase or by phosphatidylcholine:diacylglycerol cholinephosphotransferase (**Figure 2**). The latter reaction often involves PC molecules enriched in PUFAs (Lu et al., 2009). In *B. napus* the reaction is responsible for less than 20% of the PUFAs in seed oil, in contrast to about 40% in Arabidopsis (Bai et al., 2020). These data and other research (Li et al., 2006) suggest that care must be taken when assuming that *B. napus* metabolism is always identical to Arabidopsis.

Earlier studies on GPATs and genes coding for them are covered in Weselake (2005). Many GPATs can use a range of acyl-CoAs but those from oil seed species often have a preference for palmitoyl-CoA. Of various isoforms of GPATs, type 9 has been identified as important in developing seeds of Arabidopsis (Shockey et al., 2016; Singer et al., 2016a).

The next enzyme is lysophosphatidic acid acyltransferase (LPAAT) which, in *B. napus*, is the most active component in the Kennedy pathway (Perry et al., 1999). Despite this, transgenic lines with increased LPAAT activity still show enhanced lipid deposition (Woodfield et al., 2019). The enzyme has been characterised in a number of plant species (Weselake, 2005). LPAAT from *B. napus* does not effectively use lauryl-CoA or erucoyl-CoA in *in vitro* assays (Weselake, 2005), and this agrees with an absence of erucic acid from the *sn*-2 position of TAG in HEAR lines (Norton and Harris, 1983). Nevertheless, high-erucate lines of *B. oleracea* do contain appreciable erucate at the *sn*-2 position (Taylor et al., 1994). LPAAT from *B. napus* shows a preference for lysophosphatidic acid containing oleate (Oo and Huang, 1989).

The deduced amino acid sequences of plant LPAAT cDNA clones have been compared to other organisms and two conserved regions have been identified. These are box 1 (NHXXXXD) and box 2 (FP/VEGTR) (Frentzen and Wolter, 1998). Because the LPAAT from *B. napus* does not accept lauroyl-CoA or erucoyl-CoA, genetic engineering to produce high lauroyl or erucoyl oils poses a problem (see Weselake, 2002). For medium chain oils (such as cocoa butter substitutes) a medium chain thioesterase from California bay (*Umbellularia californica*) was introduced into *B. napus* (Voelker et al., 1996) together with a LPAAT from coconut which could use lauroyl-CoA (Knutzon et al., 1999). This yielded a seed oil with up to 67 mol % lauric acid. In addition, different engineering approaches for increasing saturated FAs in *B. napus* oil have been reviewed (Stoll et al., 2005). Although LEAR varieties of *B. napus* are low in saturated FAs (6-7%) there is some marketing interest in lowering this to below 3.5% and, thus, producing a ‘zero sat’ oil (Weselake et al., 2017). Low levels of saturated fatty acids have been achieved in *B. napus* (4.3 mol %) by introducing a desaturase from *Anacystis nidulans* (Shah and Weselake, 2011). Similarly, introducing a desaturase from *Caenorhabditis elegans* (that was very active with palmitate) into Arabidopsis reduced the oil content of palmitate by over 60% (Fahy et al., 2013; Weselake et al., 2017).

Typical LEAR (Canola) varieties contain around 60% oleate and this can be increased to around 85% by reducing expression of *FAD2* (Stoutjeskijk et al., 2000; Kinney et al., 2002). It may be that RNA interference could be used to silence expression of the *FAD2* gene in rapeseed, as it has in flax (Chen et al., 2015). In HEAR lines of *B. napus*, the levels of erucic acid can be increased to give oils with better industrial properties such as feedstocks for high temperature lubricants, surfactants and surface coverings. In a similar way to overcoming the substrate selectivity of *B. napus* LPAAT that was used to produce laurate oils, the LPAAT from Meadowfoam (*Limnanthes alba*) has been introduced to yield seeds synthesising appreciable trierucin (Lassner et al., 1995; Brough et al., 1996). Introduction of a yeast *sn*-2 acyltransferase into a *B. napus* HEAR line (cv. Hero) increased the erucate content of its oil from about 45% to 49-56% (Zou et al., 1997). These results were confirmed by appropriate field trials (Taylor et al., 2002) and, moreover, also revealed increased overall oil content and seed yield. In addition, there has been interest in the elongation product of erucic acid, nervonic acid, which can have nutraceutical value (Weselake et al., 2017).

The third enzyme in the Kennedy pathway is phosphatidic acid phosphatase (PAP). The enzyme was characterised from microspore cultures of *B. napus* (Weselake, 2002). Some of the activity (10-20%) was associated with microsomes but the majority was soluble (Weselake, 2005). The membrane-associated activity had properties similar to the two forms in mammalian preparations with functions in lipid synthesis or in signalling. Properties of the *B. napus* PAP and comparison with other plant PAPs are discussed in Weselake (2005).

More recent publications have expanded our knowledge of plant PAPs. Its substrate, PA, plays key roles in plant growth, development and responses to stress (Yan and Xue, 2018) in addition to its importance as a key intermediate in the Kennedy pathway. For lipid metabolism soluble PAP activity is termed PAP1 (Nakamura et al., 2009) and two forms, called PAH1 and PAH2, have been studied from Arabidopsis (Eastmond et al., 2010). PAP2 is a membrane-located PAP that also dephosphorylates other lipid phosphates. Hence, it is called lipid phosphate phosphorylase (LPP). Nine genes for LPP have been identified in Arabidopsis (Nakamura, 2013). Lipins from plants, including *B. napus*, have been confirmed as PAPs by restoring activity in yeast mutants (Mietkiewska et al., 2011). The evolutional origin of LPPs have been reviewed (Nakamura et al., 2007) while the signalling functions of plant PA are covered in (Yan and Xue, 2018; Yao et al., 2025).

The final reaction of the Kennedy pathway is that of diacylglycerol acyltransferase (DGAT). DGAT has been studied extensively in microsomal fractions from developing seeds and potential problems in some assay systems identified (Weselake, 2005). Both LEAR and HEAR lines of *B. napus* can use lauroyl-, oleoyl- and erucoyl- CoA with similar substrate specificities (Cao and Huang, 1987). Other experiments on DGAT substrate preferences in *B. napus* are described in Weselake (2005). The enzyme was solubilised from microspore-derived cultures (Little et al., 1994) and a 39 KDa polypeptide detected (Weselake et al., 1995). cDNAs for BnDGAT1 and BnDGAT2 have been cloned from microspore-derived cultures of *B. napus* cv. Jet Neuf (Nykiforuk et al., 1999, Nykiforuk et al., 2002) and their predicted proteins had molecular masses of about 57 and 40 KDa, respectively. BnDGAT1 was 96% homologous with BnDGAT2 and had 85% identity with an Arabidopsis DGAT (Weselake, 2005). The N-terminal fragment (residues 1-116) of BnDGAT1 has been expressed in *E. coli* (Weselake, 2005) and seems to be an acyl-CoA binding sequence (Nykiforuk et al., 2002). Comparative work with Arabidopsis DGAT1 suggested that the N-terminus was on the cytosolic side of the endoplasmic reticulum (Hobbs et al., 1999).

Expression of DGAT activity in developing seeds of *B. napus* shows a peak at the same time as storage oil production (Tzen et al., 1993; Weselake et al., 1993). Radiolabelling studies in developing *B. napus* (Perry and Harwood, 1993a, Perry and Harwood, 1993b) suggested that DGAT could exert significant flux control over oil accumulation, and this was confirmed by further experiments on pool sizes in the Kennedy pathway and enzyme activity measurements (Perry et al., 1999). Indeed, measurement of the flux control coefficient showed that DGAT had strong control over carbon flux into oil (Weselake et al., 2008; Tang et al., 2012) and overexpression of DGAT1 increased oil production which was also found in field trials (Taylor et al., 2009). These data allowed quantitative analysis of the role of DGAT and other enzymes involved in oil synthesis by metabolic control analysis (Harwood et al., 2013; Fell et al., 2023). Seed-specific expression of Arabidopsis DGAT1 was also able to increase seed oil content and crop yield (Jako et al., 2001) while an Arabidopsis mutant (AS11) with a reduced TAG/DAG ratio contained reduced DGAT activity due to a mutant allele in the DGAT gene (Zou et al., 1999).

Reduced DGAT activity has also been shown to have development effects in several plant species. For example, *B. napus* plants in which *DGAT1* expression was reduced using antisense-mediated knockdown resulted in the production of fewer siliques, with reduced numbers of seeds that exhibited a shrunken and wrinkled morphology and showed reduced germination rates. Flowering time was delayed by several weeks in these lines, and some flowers had larger pistils and failed to develop properly (Lock et al., 2009). A similar delay in flowering time was observed in the Arabidopsis AS11 mutant (Katavic et al., 1995), while in tobacco reduced *DGAT1* expression resulted in reduced levels of protein and seed weight, but did not adversely affect plant development (Zhang et al., 2005). Furthermore, combined loss of DGAT1 and PDAT1 resulted in the formation of sterile pollen that lacked oil bodies and embryonic defects (Zhang et al., 2009a) in Arabidopsis. These morphological abnormalities may reflect a broader function for DGAT, as indicated by its expression in many tissues that are not involved in oil accumulation and its involvement in phytohormone signaling pathways (Lu and Hills, 2002; Lu et al., 2003).

Genes encoding plant DGATs have usually been heterologously expressed in *S. cerevisiae* strain H1246 (devoid of TAG synthesis). Useful recent studies of DGATs include (Aznar-Moreno et al., 2015; Greer et al.,2015; Demski et al., 2019; Jeppson et al., 2020; Nykiferuk et al., 2002; Chen et al., 2022) and those for Arabidopsis are summarised in Chen et al. (2022). In *B. napus*, there are four forms of DGAT1 (Aznar-Moreno et al., 2015; Greer et al., 2015, Greer et al., 2016). Properties and regulation of BnDGAT1, including that by acyl-CoA, sucrose non-fermenting-1-related kinase and PA, are detailed in Chen et al. (2022). Such studies suggested that DGAT2 might be preferable to BnDGAT1 in coping with DAGs containing unusual fatty acids (Chen et al., 2022). *In vitro* assays of all four isoforms of BnDGAT1 showed that, while a range of acyl-CoAs could be used when dioleoylglycerol was the acyl acceptor, linolenyl-CoA was the best donor (Greer et al., 2016). Additional studies have used *B. napus* LEAR v HEAR lines. Two of the BnDGAT2 isoforms were effective in using erucoyl-CoA and their selectivity was increased during the breeding of HEAR lines (Demski et al., 2019). Moreover, the polypeptide region responsible for acyl-CoA specificity was later identified (Jeppson et al., 2020).

The specificity activity of DGATs can be altered by amino acid changes (Chen et al., 2022). Originally this was shown in maize (Zheng et al., 2017) but this has been expanded to the DGAT1 of *B. napus* (Xu et al., 2017) and other oilseeds (Chen et al., 2022). Depending on the substitutions concerned, different mechanisms to increase DGAT activity seem to be involved (Xu et al., 2017). Such increases in activity of high-performance variants of BnDGAT have been shown to increase oil accumulation as well as providing structure/function information (Chen et al., 2017; Xu et al., 2017).

In addition to membrane-bound DGAT1 and DGAT2 isoforms, a soluble DGAT (DGAT3) was purified and cloned from peanut cotyledons (Saha et al., 2006; Chi et al., 2014) where three isoforms were found. Similar DGAT3 enzymes have been detected in a number of other plants including Arabidopsis (Chen et al., 2022). As mentioned previously, a fourth DGAT is found in plants which is a bifunctional enzyme (wax synthase/diacylglycerol acyltransferase) important for wax ester formation. It has been characterised in Arabidopsis and several other plants (Chen et al., 2022).

The three-dimensional structure of human DGAT (from cryo-EM studies) has been reported and this enabled modelling a putative structure for the BnDGAT1 (Chen et al., 2022). The predicted model could be used to predict which residues could improve the activity of DGAT and also use a directed evolution method to enhance performance (Chen et al., 2017). Recently, the cryo-EM structure of Arabidopsis DGAT has been reported and, in addition, fatty acid regulation of the enzyme described (Liu et al., 2025).

Other properties of *B. napus* DGAT and comparison with the enzyme from other plants are detailed in Weselake (2005). Additionally, a recent thorough review about DGAT has been published (Chen et al., 2022) that covers information about the structure of DGATs, methods for assay, properties, modification to alter activity, wax synthase/DGAT and metabolic engineering to alter its role in TAG formation. The review also makes many comparisons between DGATs from bacteria, yeast, algae and mammals.

Apart from DGAT, phospholipid: diacylglycerol acyltransferase (PDAT) can generate TAG by allowing a non-acyl-CoA-dependent acylation of DAG. The acyl groups are provided by nitrogenous phosphoglycerides (phosphatidylethanolamine and, particularly, phosphatidylcholine). The PDAT reaction was first reported in plants by Dahlqvist et al. (2000) and characterised further by the same group (Ståhl et al., 2004). DGAT and PDAT have overlapping functions in Arabidopsis but it seems that PDAT is not a major contributor to oil formation in most oil seeds (Mhaske et al., 2005). Nevertheless, PDAT may be important in seeds that accumulate highly unsaturated oils or those with unusual fatty acids (Fenyk et al., 2022). *In vitro* measurements of DGAT and PDAT in *B. napus* (Tang et al., 2012; Frenyk et al., 2022) and quantitation of TAG molecular species in developing seeds (Woodfield et al., 2018) suggested that DGAT was more important than PDAT. Indeed, PDAT overexpressing lines of *B. napus* showed a small but consistent decrease in TAG accumulation, despite a several-fold increase in PDAT activity. The transgenics showed changes in the heterologous distribution of PC and TAG molecular species as revealed by MALDI-MS (Fenyk et al., 2022). These data, together with others, confirm the results of Marmon et al. (2017) that interactions of DGAT and PDAT are important in controlling both the quantitative and qualitative aspects of TAG accumulation in different oil seeds (Fenyk et al., 2022).

The acyl composition of seed TAG will be influenced by substrate selectivities of the four Kennedy pathway enzymes and, in addition, by those of PDCT, LCAT and PDAT. Furthermore, other enzymes such as phospholipases or LACS can play a role (Weselake et al., 2024). In particular, acyl re-modelling (‘acyl editing’) is important for plant tissues accumulating polyunsaturated or unusual fatty acids (Bates, 2016). *B. napus* oil is enriched in oleate but the model plant Arabidopsis (which has a distinctly different fatty acid composition) has been studied in detail regarding acyl editing (Bates, 2016; Weselake et al., 2024). In oilseed rape, sucrose-dependent 1 (SDP1) TAG lipase is present and its activity can lead to significant reductions in the oil present (Kelly et al., 2013). Other hydrolases and/or catabolic processes can also lead to losses in *B. napus* seed oil TAG (Zhou et al., 2017; Ding et al., 2019; Karunarathna et al., 2020).

Of course, once a fatty acid has been released through lipase action, it is available for beta-oxidation. A recent study of several plant tissues including a HEAR cultivar of developing *B. napus* seeds, revealed that beta-oxidation was active at all stages of development (Koley et al., 2025). The authors suggested that this observation may apply to most (or all) plant tissues that synthesise lipids. Indeed, transgenic plants where biosynthetic enzymes have enhanced activities may fail to show anticipated increases in storage oil due to increased degradation.

Once TAG has been formed on the endoplasmic reticulum, it is accumulated in oil bodies, formed by pinching off from the ER (Tzen et al., 1993; Huang, 1996: Jolivet et al., 2011). The oil bodies have a hydrophobic core surrounded by a half-unit membrane of phosphoglycerides in which proteins are embedded. The main oil body protein is oleosin but the size and composition of oil body proteins and fatty acid composition changes during seed development (Jolivet et al., 2011). For some recent general updates on oil body composition, biochemistry and function see (Chapman et al., 2012; Pye et al., 2017; Shao et al., 2019; Huang, 2019) and specifically for *B. napus* (Jolivet et al., 2009, Jolivet et al., 2011; Yin et al., 2018).

## 3 Regulation of lipid metabolism

Numerous studies have identified transcription factors (TFs) that have key roles in regulating oil biosynthesis in Arabidopsis and *B. napus*. However, it is also important to consider that oil accumulation is not only regulated at the transcriptional level, but also at post-transcriptional and post-translational levels (Kong et al., 2025).

Classical regulators, such as the LAFL (LEC1, ABI3, FUS3 and LEC2) transcription factors and WRINKLED1 (WRI1), are components in a central regulatory framework connecting seed development and oil biosynthesis (Kong et al., 2025). LAFL TFs act upstream of WRI1, a well-characterised APETALA2/ethylene-responsive element-binding transcription factor that regulates lipid accumulation (Focks and Benning, 1998; Cernac et al., 2006). It is one of the most extensively studied core TFs regulating lipid accumulation, and studies have shown that the Arabidopsis *wri1* mutant endosperm accumulated less lipid than wild type (Focks and Benning, 1998; Cernac et al., 2006). WRI1 is involved in multiple regulatory modules, and interacts with TFs and other factors such as TEOSINTE BRANCHED1/CYCLOIDEA/PROLIFERATING CELL FACTOR4 (TCP4), basic leucine zipper (bZIP) TF bZIP5, energy sensor kinase (KIN10), BTB/POZMATHs (BPMs), MEDIATOR15 (MED15), 14-3–3 protein (14-3-3), B-BOX-DOMAIN PROTEIN32 (BBX32), PHYTOCHROME-INTERACTING FACTOR4 and PIF5 (PIF4/5) and SAP AND MIZ1 DOMAIN-CONTAINING LIGASE1 (SIZ1; Kong et al., 2025 and references cited therein). Some of these interactions affect stability of WRI1 through post-transcriptional and post-translational regulation including ubiquitin-mediated degradation, phosphorylation, and sumoylation, thereby resulting in enhanced or reduced TAG accumulation (Liu et al., 2025b). These studies in Arabidopsis provide important information about the regulatory role of WRI1, although species-specific mechanisms may also exist. It is likely that the regulatory modules are more complex than currently understood, and additional proteins may form part of complex regulatory modules involving multiple proteins including master regulator TFs such as WRI1.

Several review papers have summarized the regulation of oil in plants including Arabidopsis, *B. napus* and soybean (Yang et al., 2022; Sagun et al., 2023; Weselake et al., 2024; Shen et al., 2025; Tong et al., 2025; Kong et al., 2025). Herein, we will introduce a few of the most recent updates about the regulation of lipids in *B. napus*. Han et al. (2025) mapped genetic variations controlling the expression of the key seed development and oil biosynthesis regulators, WRI1 and LAFL, in 302 rapeseed accessions at 20 and 40 days after flowering, identifying 237 expression quantitative trait loci (eQTLs) and 51 expression QTL-by-environment interactions (eQEIs; Han et al., 2025). Integrating RNA-seq, assay for transposase-accessible chromatin with next generation sequencing (ATAC-seq), and machine learning, predicted 15 candidate regulators, with multiple lines of validation. Notably, BnaC03.MYB56 was shown to activate *BnaA09.LEC1* and, along with *AGL15*, *VAL1*, and *MYB56* paralogs, co-expressed in a seed oil–related module at 20 days after flowering (DAF; Han et al., 2025). Functional tests in Arabidopsis confirmed that MYB56 functions upstream of the LEC1-WRI1 regulatory module and positively influences seed oil accumulation (Han et al., 2025). This work provides new insights into the transcriptional regulation of seed oil development and a framework for eQEI discovery.

R2R3-MYB transcription factors have also been found to regulate oil accumulation in Arabidopsis. AtMYB96 acts as a positive regulator affecting oil accumulation by activating the expression of two key genes in TAG assembly, which encode acyl-CoA:diacylglycerol acyltransferase 1 (DGAT1) and phospholipid:diacylglycerol acyltransferase 1 (PDAT1), respectively. AtMYB96 mediated TAG accumulation is independent of WRI1-regulated fatty acid biosynthesis (Lee et al., 2018). Similarly, MYB1 from *Jatropha curcas* was reported to positively regulate oil accumulation by directly binding to the DGAT1 promoter and activating its expression (Khan et al., 2019). On the other hand, AtMYB89 is a negative regulator affecting seed oil accumulation by directly inhibiting *WRI1* and other important genes for oil biosynthesis (Li et al., 2017). These examples indicate that different TFs belonging to the same family can regulate oil accumulation through different mechanisms. More recently, a study has shown that Arabidopsis plants overexpressing *AtMYB73* have altered seed oil content and fatty acid composition, driven in part by AtMYB73 directly binding and repressing the *FATTY ACID ELONGATION1* (*FAE1*) promoter (Yang et al., 2025). *AtMYB73* is induced by abscisic acid (ABA) via ABA-responsive element binding factor 2 (ABF2) binding to its promoter. The AtMYB73 protein is intrinsically disordered, forming liquid-like phase-separated condensates that influence target gene regulation (Yang et al., 2025). Together, these findings indicate that AtMYB73 integrates hormonal signaling and suggests phase-separated condensate-mediated regulation to fine-tune seed fatty acid and triacylglycerol biosynthesis.

As discussed in Section 2, it has been shown that oil accumulation can also be regulated by controlling TAG degradation (Kelly et al., 2013) through SDP lipase or other catabolic processes. Recently, a MYB-like helix-turn-helix TF, PHR1-like 7 (PHL7), was reported in Arabidopsis (Tang et al., 2025). The *phl7* knockout mutant displayed an approximately 10% increase in seed oil content, while overexpression of *AtPHL7* resulted in a 8-13% decrease (Tang et al., 2025). Further analyses showed that AtPHL7 binds to the promoter of the TAG degradation gene *SUGAR-DEPENDENT1 (SDP1)* and promotes *SDP1* expression (Tang et al., 2025). Moreover, the authors found that phosphatidic acid (PA) binds to AtPHL7 and inhibits the binding to the *SDP1* promoter (Tang et al., 2025). These results indicate that the interaction between the important metabolic intermediate PA and AtPHL7 regulates TAG degradation and seed oil accumulation. Whether a similar mechanism exists in *Brassica napus* remains to be determined.

When overexpressing master transcription regulators of fatty acid biosynthesis *Brassica napus* LEAFY COTYLEDON1 (LEC1) and LEC1-LIKE (L1L), using the seed-specific napin promoter, plants showed increased seed fatty acid by 2-20% (Tan et al., 2011). Importantly, these transgenic plants do not have negative effects on plant growth and development as seen when the same genes are driven by the CaMV35S promoter (Tan et al., 2011). These results indicate the importance of seed-specific promoters and revealed the effective promoter regions for regulating oil accumulation. Some other TFs such as LEC2, FUSCA3 (FUS3), ABSCISIC ACID INSENSITIVE3 (ABI3), Dof zinc finger protein (DOF) (ZIP123), and Zinc finger transcription factor (ZF) have also been found to regulate oil accumulation. Please refer to recent review papers for more information (Song et al., 2022; Yang et al., 2022; Sagun et al., 2023; Weselake et al., 2024; Gao et al., 2025; Kong et al., 2025; Shen et al., 2025; Tong et al., 2025).

Genome- and transcriptome-wide association studies (GWAS and TWAS) are useful tools to identify quantitative trait loci (QTLs) that regulate seed oil content (SOC) in *B. napus*. For instance, using GWAS and TWAS in 505 *B. napus* inbred lines across eight environments, allowed a study to map robust SOC QTLs, assess their effects and breeding selection, and identified 692 genes and four seed gene modules associated with SOC. A multi-omics prioritization framework (POCKET) pinpointed causal candidates within QTLs, leading to the discovery of homologous genes *Probable methyltransferases* (*BnPMT6s*) that negatively regulate SOC and were validated experimentally (Tang et al., 2021). These results provide rich genetic resources and insights into the complex regulation of seed oil accumulation in *B. napus* and other oil crops.

The complex genetics of *B. napus*, attributable to its allopolyploid genome arising from a natural hybridization between *B. rapa* (AA, 2n=20) and *B. oleracea* (CC, 2n=18), represents a major challenge to the application of GWAS and TWAS approaches. Furthermore, the allotetraploid genome presents complications for generating genetically stable populations following hybridization of different *B. napus* varieties, and for genetic modification strategies such as gene editing of specific loci in the constituent sub-genomes. Considerable advances have been made in producing accurate *B. napus* reference genome sequences to enable the identification of loci associated with agronomically important traits, in particular the work by Song et al. (2020) that used multiple different DNA sequencing technologies to produce genome sequences for eight different *B. napus* accessions. The data were used to generate a pan-genome sequence that was used to identify single-nucleotide polymorphisms (SNPs), insertions and deletions (Indels), structural variations (SVs), copy number variations (CNVs) and presence and absence variations (PAVs) that relate to important morphological traits such as seed weight, silique size and flowering time (Song et al., 2020). This work also enabled a more complete analysis of *B. napus* phylogeny, revealing conserved and dispensable gene clusters amongst the different accessions, and further work has used pan-genome sequences to investigate the ancestry and evolution of the *Brassica* genus A, B and C sub-genomes (He et al., 2021).

Previous studies have reported the gene expression profiles of *B. napus* seed subregions such as the seed coat, endosperm and embryo through transcriptomics analysis (Liao et al., 2019; Ziegler et al., 2019; Gao et al., 2022; Wei et al., 2022). However, as *B. napus* contains diploid genomes from both *Brassica rapa* (An subgenome) and *Brassica oleracea* (Cn subgenome), it remains unknown whether there is subgenome bias relating to the control of *B. napus* seed development. To address this question, researchers built a comprehensive spatial–temporal transcriptome atlas of *B. napus* seeds, covering maternal seed coat and filial embryo/endosperm subregions, and revealed a pervasive bias toward the Cn subgenome (Ziegler et al., 2025). This bias is strongest early in development and particularly pronounced in maternal tissues; notably, the chalazal pole shows a distinct transcriptome, suggesting unique developmental roles (Ziegler et al., 2025). In mature seeds, storage-related genes dominate, especially in the embryo, while key homoeologue pairs involved in seed development display low transcriptional bias, providing a nuanced view of polyploid gene regulation across seed development (Ziegler et al., 2025). This study offers an important synthesis of polyploid transcriptomics in seed biology and provides a comprehensive spatiotemporal overview of the *B. napus* gene expression landscape.

Histone modifications are reversible chemical changes (e.g., acetylation, methylation, phosphorylation, ubiquitination, sumoylation) of histone proteins that alter chromatin structure and regulate gene expression, constituting a major form of epigenetic regulation. Until recently, it was unclear whether histone modifications are present in dry seeds of *B. napus*, and if so, what their types and functions are (Zhang et al., 2024). Although chromatin immunoprecipitation with next-generation sequencing (ChIP-seq) is the gold standard for identifying genome-wide histone modifications and chromatin-associated protein binding sites, it has been difficult to apply to economically important plant organs with sturdy cell walls and complex constituents (Zhang et al., 2024). Recently, an advanced ChIP (aChIP) was developed that efficiently isolates chromatin, enabling precise profiling of histone modifications and transcription factor/chromatin-modifying enzyme binding across 14 different economically important plant organs, including seeds, flowers and fruits, and increasing sensitivity to reveal many novel modification sites compared with prior methods (Zhang et al., 2024). Notably, it has been applied to dry seeds of *B. napus*, mapping the histone landscapes and underscoring the complex roles of chromatin dynamics in seed dormancy and germination (Zhang et al., 2024). Together, this study establishes aChIP as a powerful, broadly applicable tool for comprehensive chromatin profiling in challenging plant tissues.

## 4 Spatiotemporal aspects of oil accumulation

Advances in technologies such as genome-wide association studies (GWAS), comparative functional genomics, transcriptomics, and quantitative trait loci (QTL) analysis have led to the identification and characterisation of numerous genes critical for regulating oil accumulation (Song et al., 2022; Gu et al., 2024; Zhang et al., 2025). Various strategies, including the modulation of key genes involved in lipid biosynthesis, carbon partitioning, fatty acid composition and transcriptional regulation have been employed to enhance oil accumulation (Weselake et al., 2024). Recent progress in biotechnological approaches targeting these genes and pathways has been extensively reviewed (Yang et al., 2022; Sagun et al., 2023; Weselake et al., 2024; Gao et al., 2025; Shen et al., 2025; Tong et al., 2025; Wei et al., 2025). In this section, we will explore the latest advancements in modifying oil accumulation.

Traditionally, the seed embryo was considered the primary determinant of oil accumulation, leading to limited research on the role of the seed coat. However, recent studies have identified key genes highly expressed in the seed coat that significantly influence oil production. For example, *BnaMYB52*, which is strongly expressed in the seed coat but not the embryo during seed development, was discovered through genome-wide association studies across *B. napus* accessions (Ye et al., 2025). Knocking out *BnaMYB52* in *B. napus* reduced seed coat content by ~8% while increasing seed oil content (SOC) by ~13%. Conversely, overexpressing *BnaMYB52* produced the opposite effects (Ye et al., 2025). Further investigations revealed that *BnaMYB52* modulates seed coat thickness and SOC by activating *BnaPMEI14* (encoding pectin methylesterase inhibitor 14) and *BnaBAN* (*BANYULS*, encoding anthocyanidin reductase; Ye et al., 2025). In Arabidopsis *ban* and *pmei14* mutants, seed coat thickness decreased by ~16-23% and ~13-22%, respectively, while SOC increased by ~5% and ~7% (Ye et al., 2025). These findings demonstrate that *PMEI14* and *BAN* promote seed coat thickness but negatively regulate SOC. This study provides a novel strategy for enhancing oil accumulation by targeting seed coat-specific genes and modifying seed coat thickness.

Yellow seed coat colour is strongly correlated with high SOC and low seed lignocellulose content (Li et al., 2025). A rare dominant allele, *DYSOC1* (dominant gene of yellow seed coat colour and improved seed oil content 1), was identified through QTL fine-mapping and multi-omics analysis and found to significantly influence SOC (Li et al., 2025). Transgenic *B. napus* plants overexpressing *DYSOC1* exhibited a yellow seed coat colour, a ~10% increase in SOC, and a ~36% reduction in seed coat thickness (Li et al., 2025). Conversely, *DYSOC1* knockout lines displayed a darker seed coat colour, a ~7% decrease in SOC, and a ~36% increase in seed coat thickness (Li et al., 2025). Other flavonoid biosynthetic pathway genes such as *TRANSPARENT TESTA1 (TT1)*, *BrTT8* and *TRANSPARENT TESTA GLABRA1 (TTG1)* from *B. rapa*, have been reported to affect yellow seed coat colour previously (Li et al., 2012). A large insertion in bHLH transcription factor *BrTT8* resulting in a yellow seed coat in *B. rapa* (Zhang et al., 2009b; Li et al., 2012, Wang et al., 2017). However, in *B. juncea*, *BjTT8* was found to be the only gene affecting the yellow seed coat trait that coevolved with a MADS-box gene SEEDSTICK (STK) to coordinately affect seed size, seed coat proportion and oil accumulation (Qian et al., 2025). Together, these findings highlight strategies for enhancing oil accumulation by manipulating the trait of yellow seed coat colour in *B. napus*.

Growing evidence demonstrates an interplay between flavonoid biosynthesis and oil accumulation pathways and there is a dynamic homeostasis between these two pathways (Lécureuil et al., 2025; Qian et al., 2025). This suggests that modulating flavonoid biosynthesis genes could influence oil accumulation. Indeed, *TRANSPARENT TESTA7* (*TT7*) was identified as a regulator of oil accumulation in Arabidopsis (Lécureuil et al., 2025). The Arabidopsis *tt7* mutant exhibited reduced SOC but accumulated higher levels of kaempferol-3-O-rhamnoside (Lécureuil et al., 2025). These findings suggest that overexpression of *TT7* in *B. napus* might enhance SOC.

Genome-wide association studies conducted across different developmental stages of *B. napus* seeds identified *BnaMYB56*’s role as a novel transcription factor (Han et al., 2025). Mutants of its Arabidopsis homolog showed significantly reduced SOC, confirming *MYB56*’s role as a positive regulator of oil accumulation (Han et al., 2025). This finding establishes *BnaMYB56* as a promising molecular target for enhancing oil accumulation in *B. napus*. AtAPETALA2 (AP2), a member of the AP2/ETHYLENE RESPONSIVE ELEMENT BINDING PROTEIN TF family, plays essential roles in floral meristem and organ development in Arabidopsis (Wang et al., 2026). The Arabidopsis *ap2* mutant produces larger seeds with increased weight and higher oil content per seed, though the overall seed oil yield decreases due to reduced seed production (Jofuku et al., 2005; Ohto et al., 2005). In *B. napus*, knockdown or knockout of all four *BnAP2* genes resulted in undesirable phenotypes including abnormal floral organs and decreased seed yield (Yan et al., 2012). However, specific targeted knockout of *BnaA01.AP2* using CRISPR/Cas9 technology significantly increased both seed yield and oil content without compromising plant growth or yield penalty in *B. napus* (Wang et al., 2026).

Seed yield represents a crucial agronomic trait that directly impacts total seed oil accumulation. Increased seed production enables greater overall oil yield. For example, overexpression of the cytokinin-related gene *Adenine Phosphoribosyltransferase 5* (*BnaC9.APT5*) in *B. napus* increased seed number per silique by 11% (Cui et al., 2026). Further analysis showed that this effect resulted from a 48-bp deletion in the *BnaC9.APT5* promoter region (Cui et al., 2026). These findings demonstrate an alternative approach to modulating oil accumulation through seed yield regulation.

## 5 Environmental effects on rapeseed oil production

Environmental effects can be divided broadly into abiotic and biotic factors. *B. napus* can be affected by a wide range of pests and diseases (**Table 3**). Such biotic factors will vary depending on the area studied, whether one is considering spring or winter varieties of *B. napus* and the prevailing climatic conditions. A common foliar disease is light leaf spot (*Pyrenopeziza brassiae*), while Sclerotinia (*Sclerotinia sclerotiorum*) is a soil-borne disease that affects many crops, while clubroot (*Plasmodiophora brassicae*) attacks all brassicas. Often the prevalence of fungal diseases can be alleviated by crop rotation and/or simple fungicide treatment.

**Table 3:** Environmental stress effects on rapeseed oil production.

Biotic stress	Examples	Effect on content
Diseases	Leaf spot	Decrease
	Sclerotinia	Decrease
Pests	Pollen beetle	Decrease
	Rape winter stem beetle	Decrease

Abiotic stress	Examples	Effect on content
	High temperature	Decrease
	Low temperature	Decrease
	Drought	Decrease
	Salinity	Decrease
	High light	Increase
	Low light	Decrease

There are numerous oilseed rape pests which can cause significant damage and losses to crops. These include slugs, cabbage stem flea beetle (*Psylliodes chrysocephala*), rape winter stem weevil (*Ceutorhynchus picitarsis*), pollen beetle (*Meligethes* spp.) and turnip sawfly (*Athalia rosae*). Overall global crop losses due to diseases or insect attack are estimated to be around 20% although different crops and/or various conditions can show higher reductions (Wu and Ye, 2020).

For the last two decades, our knowledge of how plants try to defend themselves from disease or insect attack has gradually advanced. Two major defence strategies have been identified: the salicylic acid (SA) and jasmonic acid (JA) signalling pathways. These pathways often work synergistically to induce defence responses but they may be antagonistic (Kachroo et al., 2001). JA is produced from alpha-linolenic acid but a specific stearoyl-ACP desaturase gene was shown to be utilised in responses to *Botrytis cinerea* infection (Kachroo et al., 2001). The SA- and JA-dependent defence pathways interact and their crosstalk can be modulated by a regulatory protein (NPR1) in Arabidopsis (Spoel et al., 2003). The crosstalk was explored further and it was noted that biotrophic pathogens (that feed and reproduce on live host cells) induce the SA defence system while necrotrophic pathogens (that kill host cells for reproduction and nutrition) activate the JA defence (Spoel et al., 2007). Examples of a biotrophic pathogen (*Pseudomonas syringae*) and a necrotrophic organism (*Alternaria brassicicola*) allowed Macioszek et al. (2020) to confirm the proposed crosstalk in *Brassica oleracea* leaves.

The involvement of stearoyl-ACP desaturase in the SA-defence pathway has been studied further in Arabidopsis and soybean which showed enhanced resistance to multiple pathogens (Kachroo et al., 2008). This subject has been explored more recently and showed that fatty acid metabolism adapts to viral infections in crop plants (Jiang et al., 2025a). Indeed, the overall topic of fatty acid-derived signals in plant defence has been reviewed (Kachroo and Kachroo, 2009). The role of the JA-defence pathway against herbivores and pathogens has been expanded recently to include viruses (Wu and Ye, 2020) and this review has a useful list of pathogens and insects of particular relevance. Although the SA and JA defence pathways are the most important for protection against biotic attack, other compounds, such as abscisic acid or brassinosteroids, may also play a role (Zhao and Li, 2021). Moreover, the lipids of pathogens themselves can play a significant role in their crosstalk with plants (Cavaco et al., 2021).

In a review about improving *Brassica napus* lines for increased oil content, a notable comment was that disease resistance to *Sclerotina* was an important trait that had been achieved to some extent (Hua et al., 2016). *S. sclerotiorum* is a necrotrophic fungus which causes an increasing problem in certain areas and, because fungicides are not always effective, development of resistant oilseed rape is a primary focus to mitigate the disease (Xu et al., 2018). BnA07.WRKY40 is a positive regulator in response to infection and affects several important downstream target genes. Moreover, knockout of these genes increased susceptibility to *S. sclerotiorum*, thus confirming the molecular mechanism by which BnA07.WRKY40 regulates the defence response of *B. napus* to infection (Lin et al., 2025).

For a useful summary of the many abiotic factors affecting oil crop production see Singer et al. (2016b). One of the most studied abiotic environmental factors is temperature. Higher environmental temperatures can have an environmental impact as noted by Singer et al. (2016b) when a warm growing season in Canada reduced *B. napus* oil content significantly. For canola varieties, night temperatures during early development have been shown to correlate inversely with oil content (Pritchard et al., 2000). In general, studies with a number of oil crops both in the lab and in field trials show that cooler temperatures, especially during the seed filling period, lead to higher oil yields. However, it should be noted that not all crops behave the same and, in a classic study Canvin (1965) noted that while higher oil contents were produced by lower growth temperatures for rape and flax, no change was found for castor bean or safflower. For oilseed rape a continuous decrease was observed for increases in growth temperature in the range 10-27°C (Canvin, 1965). He also noted a decrease in the PUFA proportions in seed oil at higher temperatures, which is a common observation for most plants (Wolf et al., 1982; Schulte et al., 2013). Often it has been speculated that the desaturases responsible for PUFA formation are sensitive to higher temperatures but, since they are aerobic, the supply of oxygen may be critical (Harris and James, 1969). This reason has been examined further in a non-plant organism (*Acanthamoeba*) that synthesises linoleic acid in response to low temperatures (Jones et al., 1993). However, in this case a multitude of factors appear to be involved (Avery et al., 1994) with new protein synthesis being critical (Jones et al., 1993). Nevertheless, oxygen solubility can be demonstrated to be solely responsible for desaturase activity in *A. castellanii* (Avery et al., 1996). In addition, high temperatures cause a down regulation of FAD2 expression in a number of plants (Byfield and Upchurch, 2007) as summarized in Singer et al. (2016b). Despite this, a cultivar of *B. napus* has been identified which has higher oil levels at higher growth temperatures, perhaps because of an increased expression of FAD2 and FAD3 and also of DGAT1 (Zhu et al., 2012).

In contrast to higher growth temperatures, *B. napus* has to be able to tolerate low temperatures, particularly for winter-sown varieties. This factor is also a major constraint for expanding areas which can be planted. Low temperature tolerance has been examined in *B. napus* (Asghari et al., 2007; Huang et al., 2018) for QTL markers. The subject has been developed recently using a combination of techniques. Inbred lines which were cold-tolerant or cold-sensitive were used and co-expression analysis utilised. Seven candidate genes induced by cold temperature treatments were identified and could be useful for future breeding (Qin et al., 2022). In rice and a cold-resistant grass (*Campeiostachys nutans*), their resistance is due to an increase in alpha-linolenic acid released by phospholipase A1 (CnPLA1) activity. Cold tolerance was improved by maintaining redox homeostasis and activating JA biosynthesis. A transcription factor, CnERF109 was shown to regulate cold tolerance by binding to the *CnPLA1* promoter, thus activating its transcription and hence playing a pivotal role in alpha-linolenic acid levels and cold adaptation (Jiang et al., 2025b). Together with data from other oilseeds, such results give a useful background for breeding cold-tolerant rapeseed lines.

As an extension to the above studies, Fu et al. (2009) studied oil production in *B. napus* at different altitudes in various field stations varying in altitude from sea level to 3658m. Unfortunately, they did not record temperatures or, indeed, average weather conditions at the three stations used where ambient temperatures would have been critical. They used a high oil *B. napus* variety which yielded 46% oil at sea level but 53-54% oil at 2261m or 3658m (Fu et al., 2009). Extrapolating that the higher elevations would have had lower temperatures, the results agree with the effects of growth temperatures discussed above. This conclusion is reinforced by the results of experiments with two near-isogenic *B. napus* lines where one had a higher seed oil content than the other (Zhu et al., 2012) and raised growth temperatures reduced the oil content and PUFA proportions in both. They used a combination of different light periods and temperatures and analysed the expression of genes under these conditions which revealed a number of genes important for lipid metabolism (Zhu et al., 2012). Combining different conditions for examining climate change is very important and holistic approaches are strongly encouraged (Martinez-Luscher et al., 2025). Indeed, the first publications examining climate change alterations on lipid content and metabolism were made some 30 years ago (Williams et al., 1994, Williams et al., 1995). This work looked at combinations of raised temperature and CO_2_ under different nitrogen fertilizer inputs. Increased CO_2_ as recorded in climate change, can itself have noticeable actions on lipid metabolism and physiological development (Robertson et al., 1995). Using combinations of changed environmental parameters to examine the effects of climate change have been discussed recently (Reed et al., 2022; Fan et al., 2025).

One change brought about by climate change is the increase in drought. On average, water shortage prevalent during drought reduces oil content and the proportion of PUFAs in a variety of oil crops (Pritchard et al., 2000; Talha and Osman, 1974; Rotundo and Westgate, 2009; Rebey et al., 2012). A particular transcription factor has been shown to enhance resistance to drought in Arabidopsis (Lee et al., 2019). Interestingly, increased expression of DGAT in *B. napus* (Weselake et al., 2008) allowed plants to overcome impacts of drought in summer-grown field studies (Taylor et al., 2009). This revealed the possibility that new lines of *B. napus* could be resistant to climate change.

Another stress encountered under field conditions is salinity and this aspect is of interest in trying to increase areas for crop planting. Salinity stress tends to cause decreases in seed oil content in the same way as drought or increased growth temperatures. Again, a reduction in the proportion of PUFAs in the seed oil is also found. These results have been observed in a variety of oil crops (Singer et al., 2016b) which include Arabidopsis and Crambe. The effect of salinity stress has been examined for spatial distributions in barley roots using three versions of mass spectrometry (see section 6). The data showed distinct distributions due to salinity in ions and lipids (especially PC species; Sarabia et al., 2018). Salt-tolerant *B. napus* has been produced where sodium is concentrated in leaves and this did not affect seed set or oil accumulation. Indeed, hardly any effects on lipid metabolism were found (Zhang et al., 2001). The successful engineering of *B. napus* is important because it has been estimated that around 25% of total irrigated acreage in the world has been damaged by salt (Ghassemi et al., 1995), and resistant crops would be extremely useful.

For all crops, light is a pre-requisite for photosynthesis and, hence, development of their yields. In several oilseeds, including soybean and oilseed rape, their developing seeds are green and contain the necessary enzymes for photosynthesis which can supply appreciable carbon for triacylglycerol production. Thus, solar radiation can have an important effect on oil content during seed filling (e.g. Goffman et al., 2005; Li et al., 2006). Seed development in *B. napus* is affected by various combinations of day length with low temperatures. As expected, low temperature growth increased PUFA levels. In addition, short days reduced total oil accumulation and the authors also reported co-expression of genes for several Kennedy pathway enzymes (Vuorinen et al., 2014) as well as confirming the conclusion that PDAT was not important for overall TAG production in *B. napus* (see Woodfield et al., 2018).

## 6 Use of MALDI-MSI aids understanding of spatial and temporal differences in plants

Mass spectrometry imaging (MSI) is a powerful technique which can be applied to the analysis of both the localisation and quantitation of metabolites *in situ* (Horn and Chapman, 2014). It has been used to examine lipids (amongst other metabolites) for over a decade (Horn and Chapman, 2024). For lipids, a general publication about techniques with examples from Arabidopsis (Lee et al., 2012) and a detailed report of oilseeds from cotton (Horn et al., 2012) were made. A major attraction of MSI is that it examines metabolites *in situ* and its non-invasive approach allows accumulation of information in a spatial way which can be extended to long time periods. Two recent reviews have discussed different techniques and pointed out saliant advantages compared to other methods. For lipids, matrix-assisted laser/desorption ionisation (MALDI-MSI) is the usual method used and typical workflows are shown in Borisjuk et al. (2023) and Horn and Chapman (2024). A detailed description of the methods, as applied to plant lipid metabolites, is given in Sturtevant et al. (2021).

**Figure 3:**
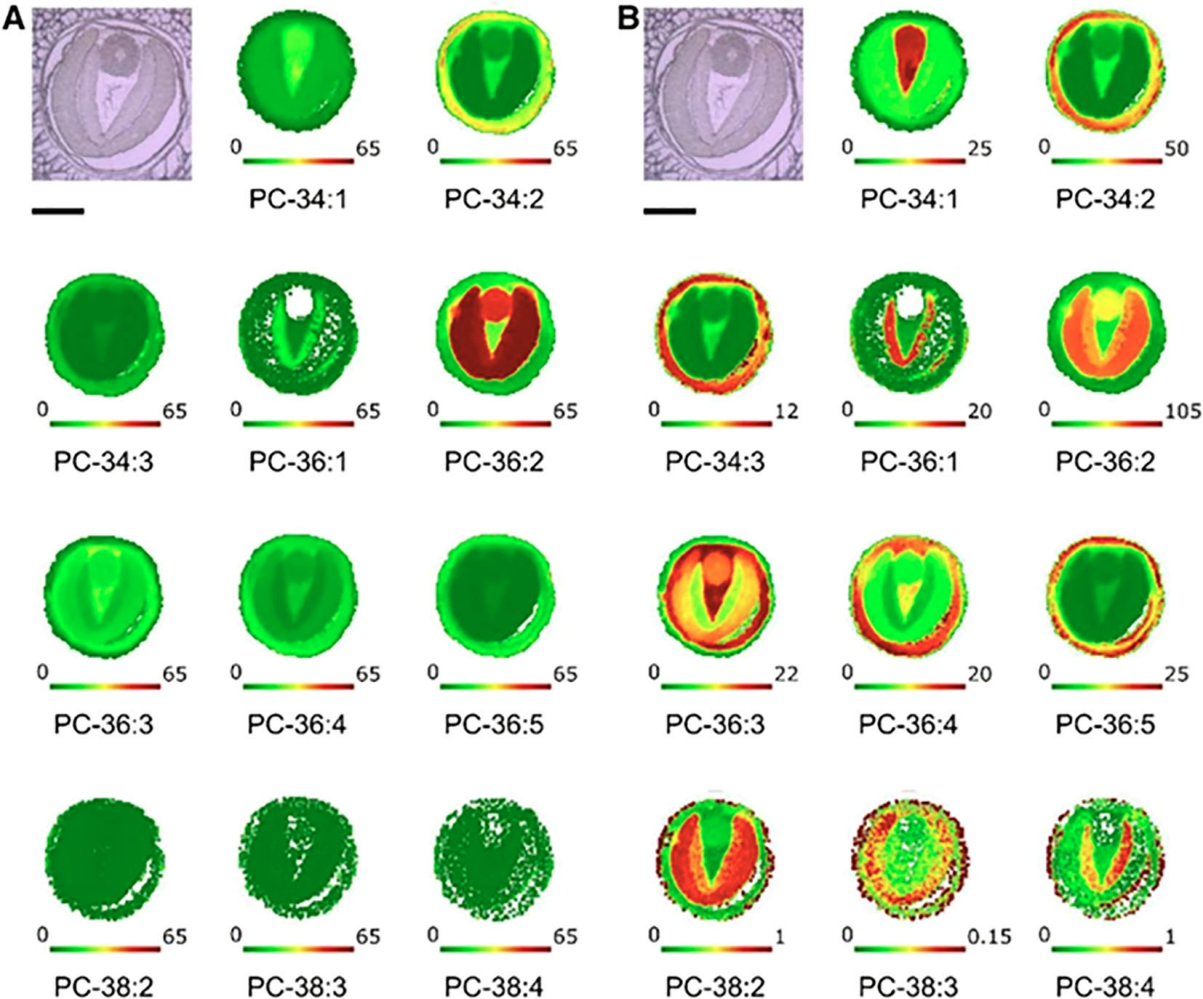
Imaging of selected PC molecular species in oilseed rape seeds at 27 DAF. Bright -field images of seed cross sections at 27 DAF are shown at top left of each part. Bars = 1 mm. The distributions of selected PC molecular species are shown with a fixed mol % to show absolute distribution profiles **(A)** and an adjusted mol % to show relative distribution profiles **(B)**. Below each image is a color scale bar, with green and red representing low and high levels, respectively. Numbers at either end of the colored bar represent the scale of that image. Each image also is labeled with the total number of acyl carbons and double bonds (e.g. PC-34:1). Reproduced from Woodfield et al. (2017).

When MSI was first applied to plant lipids, the spatial heterogeneity of metabolites was revealed. This applied to both membrane lipids (such as PC) as well as storage compounds (TAG). Examination of sections of *B. napus* seeds showed that the spatial distribution of such metabolites was similarly heterogenous. Indeed, molecular species of both PC and TAG showed significantly different compositions in the various seed tissue types (Woodfield et al., 2017b). An example of the distinct distribution of molecular species of PC is shown in **Figure 3**. In this figure both the absolute distribution profiles and the relative distribution of PC are shown within the seeds examined 27 days after flowering at the mid-point of oil accumulation. To avoid losing the distributions of the highest and lowest abundant molecular species, the scale was adjusted for the maximum ion intensity in each image. For individual tissues, the seed coat/aleurone layer contained high amounts of PC species with PUFAs while the embryonic axis had the highest relative amounts of PC species with palmitic acid. One notable feature that the MALDI-MSI revealed concerned the inner and outer cotyledons. Although these are functionally identical, their PC species were notably different (**Figure 3**). This can be seen most clearly for PC-36:1. PC-36:2 and the most prevalent species PC-38:2.

**Figure 4:**
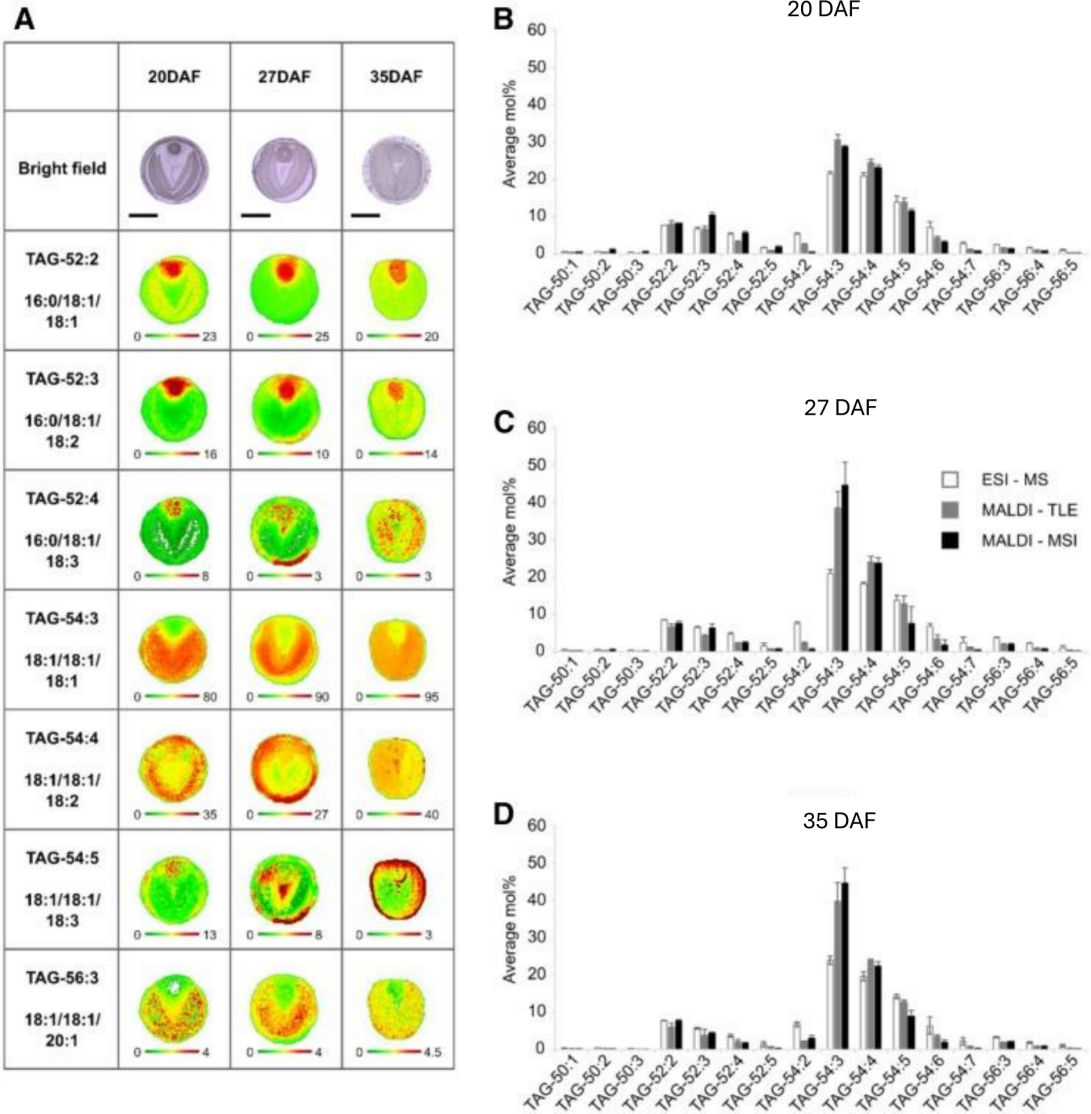
Imaging and quantification of selected TAG molecular species in oilseed rape seeds at three developmental stages. **(A)** Bright-field images of seed cross sections (top of each column; bars = 1 mm) and relative distribution patterns of selected TAG species at three time points, 20, 27, and 35 DAF (one developmental stage per column) are shown. Below each image is a color scale bar, with green and red representing low and high levels, respectively. Numbers at either end of the colored bar represent the scale of that image. TAG species with total number of acyl carbons and number of double bonds, along with the most likely acyl combination (based on MS data) below, are indicated at left. **(B–D)** Quantification of total molecular species by ESI-MS (white; *n* = 3 independent extractions), MALDI-MS of TLE (gray; *n* = 3 independent extractions), and MALDI-MSI (black; *n* = 3 sections from three different seeds) is compared at three developmental stages. Quantification in lipid extracts was based on internal standards. TLE= total lipid extract. Values shown are means ± sd. Reproduced from Woodfield et al. (2017).

Another advantage of MSI is that it allows an examination of temporal mapping of changes in metabolism, such as during development of seeds. In **Figure 4** the alterations in TAG molecular species are shown during maturation of *B. napus* seeds (Woodfield et al., 2017b). On the whole, the main storage lipid (TAG) molecular species did not change much in their % composition during development from the beginning (20 DAF) to the end (35 DAF) of seed oil accumulation. However, MALDI-MSI revealed some notable changes during this time-period. For example, TAG-54:3 and TAG-54:5 distributions altered during development (**Figure 4**). For PC molecular species the distributions were changed even more (Woodfield et al., 2017) with, for example, PC-34:2 which was located mainly in the seed coat/aleurone layer up to 27 DAF was mainly found in the embryonic axis by 35 DAF.

**Figure 5:**
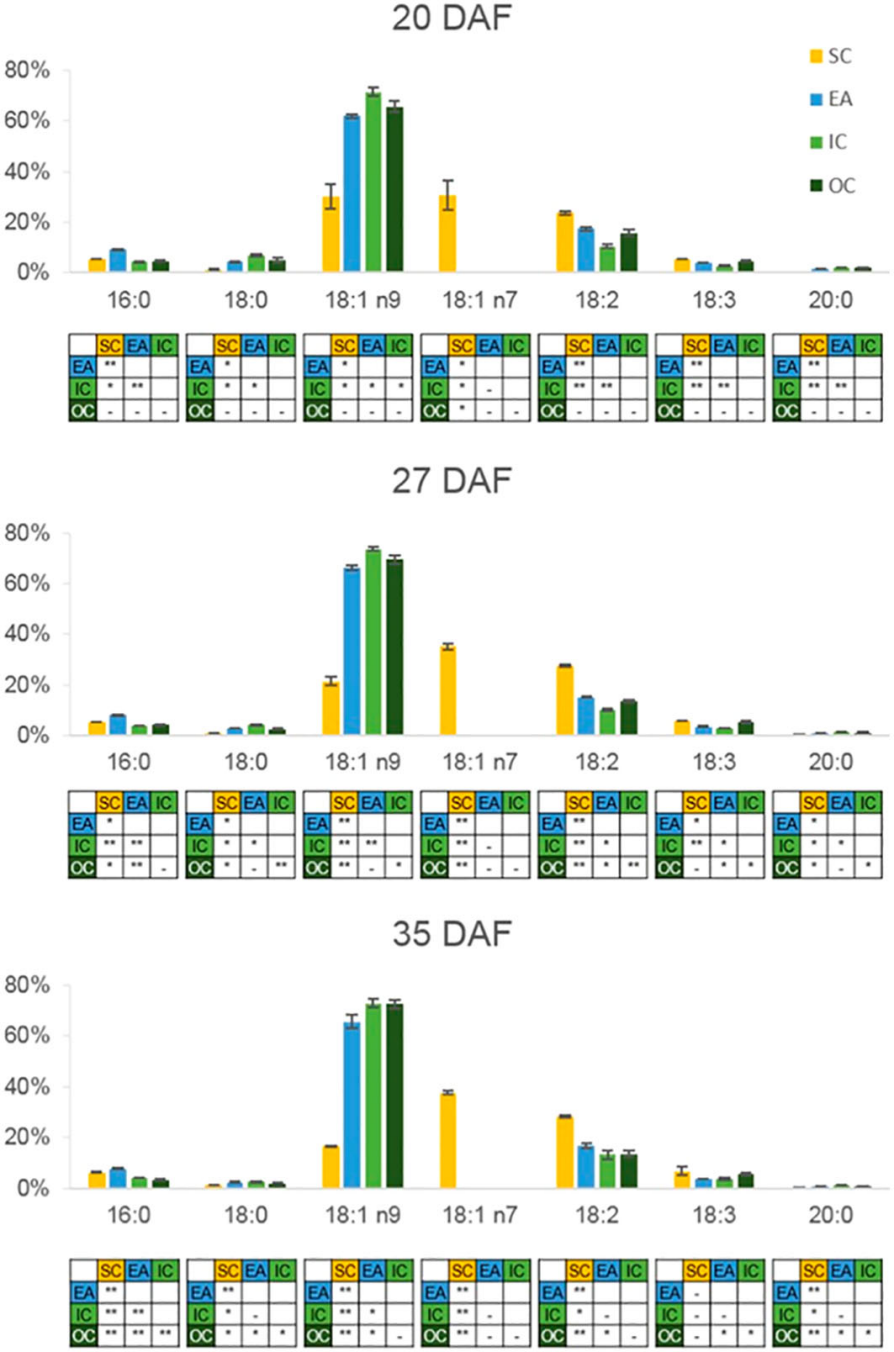
Fatty acid composition of TAG in dissected seed tissues at three developmental stages. Tissues analyzed were the seed coat/aleurone layer (SC), embryonic axis (EA), inner cotyledon (IC), and outer cotyledon (OC). Seeds were sampled at 20, 27, and 35 DAF. Values are means ± sd (*n* = 3). Tables below each graph show the statistical analysis of the fatty acid in the column immediately above: *P < 0.05; **P < 0.001; –, no significant difference. Reproduced from Woodfield et al. (2017).

There are some limitations with MALDI-MSI and, therefore, it is prudent to validate data accumulated by using a different analytical method to examine lipid content and composition. This was done by Woodfield et al. (2017), in particular to examine different component tissue types by micro-dissection. This method was able to confirm the MALDI-MSI findings and, in addition, drew attention to the presence of omega7-octadecenoate exclusively in the *B. napus* seed coat/aleurone layer. This was found for both membrane (PC) and storage (TAG) lipids (**Figure 5**) and revealed another example of the benefit of MALDI-MSI in revealing intricacies of metabolism. These data suggested an importance for the location of delta-9 desaturases (similar to Arabidopsis AAD2 and AAD3; Cahoon and Shanklin, 2000; Nguyen et al., 2010) in the aleurone layer of *B. napus* (Woodfield et al., 2017).

The use of MALDI-MSI to complement traditional biochemical studies of metabolism is an important advantage. Another example is in the work of Liao et al. (2022). Here, *B. napus* lines which were overexpressed in *GPAT, LPAAT* or *PDAT* using various heterologous transgenes were employed. The transgenic lines were examined for lipid profiles compared to wild type DH12075 (a canola or LEAR variety) plants. Distinct changes in the spatial distribution and abundance of membrane (PC) as well as storage (TAG) species were found, particularly in the LPAAT-OE and PDAT-OE lines. These data were found despite little change in seed size or overall fatty acid composition of seeds in the LPAAT-OE lines (or the GPAT-OE lines) and emphasise the ability of MALDI-MSI to reveal subtle alterations in metabolism that would not necessarily be noticed by conventional biochemistry.

As mentioned in section 2, there has been considerable interest in the relative contributions of DGAT and PDAT in forming the TAG of seed oils. There seems little doubt that PDAT is important in seeds which accumulate significant amounts of PUFAs or ‘unusual’ FAs (such as ricinoleic acid in castor bean). However, in *B. napus* evidence from enzyme activity quantitation (Tang et al., 2012) or from analysis of TAG molecular species during seed oil accumulation (Woodfield et al., 2018) suggested that DGAT was more important. In order to provide more data, further experiments with a LEAR-line of *B. napus* which overexpressed Arabidopsis *PDAT1* were conducted (Fenyk et al., 2022). Seeds from independently generated PDAT-OE lines showed changed fatty acid compositions as well as a small decrease in oil accumulated despite a several-fold increase in PDAT activity. These data indicated that PDAT exerted a small, negative control over TAG biosynthesis and agreed with other results showing that DGAT was the main enzyme responsible for TAG accumulation in *B. napus*. Simultaneous MALDI-MSI analysis revealed that the PDAT-OE lines showed tissue specific changes in the molecular species distributions of PC and TAG which would not have been revealed by conventional lipid extraction and analysis. In summary, PDAT overexpression caused a shift in lipid composition and an obvious change to the heterologous lipid distribution commonly observed in oil seeds (Fenyk et al., 2022).

The relative contributions of DGAT and PDAT for TAG formation has been examined in another oilseed crop, *Camelina sativa* (Marmon et al., 2017). Here various combinations of transgene over-expression and gene silencing were combined with fatty acid, acyl-CoA and complex lipid (PC, TAG) analysis. MALDI-MSI was able to supplement the basic quantitation and, for example, was able to reveal that DGAT activity was strongest in cotyledons, in confirmation of some previous data from Horn et al. (2013). In addition, it was noted that PDAT overexpression caused an increase in linoleate but not linolenate, in contrast to PDAT overexpression in Arabidopsis. A possible complication that was raised in the work of Marmon et al. (2017) was that a napin promoter was used and this can have a preferential effect on the cotyledon which, in turn could bias the results seen. This raises an important consideration to be born in mind when evaluating detailed spatial data as revealed by MALDI-MSI.

As described above, not only is MALDI-MSI vital for delineating spatial and temporal shifts in lipids, it can also give important information to elucidate how metabolism is changed and regulated within various circumstances. For example, Lu et al. (2018) examined high- and low-oil *B. napus* seeds. The MALDI-MSI data were compared to tissue-specific transcriptome analysis and implied that transcriptome regulation influenced the heterogenous distribution of lipids within seeds and emphasized the importance of spatial information in this context.

While the use of stable isotope labelling (Allen et al., 2015; Allen, 2016) and mass spectrometry imaging (Horn and Chapman, 2024) have been used separately to analyse plant lipidomics, their combination can offer advanced resolution of metabolic pathways. However, this approach has technical challenges that must be resolved. Romsdahl et al. (2021) describe some of these for PC analysis in the embryos of two oilseed crops (*Camelina sativa, Thlaspi arvense).* Other recent reviews for MALDI-MSI in plants (Ventura et al., 2025) and stable isotope labelling using mass spectroscopy (Anagho-Mattanovich et al., 2026) will be found useful.

MALDI-MSI has also been used to add information to the role of lipids in autophagy where PUFA-containing or very long chain lipids were implicated (Mugume et al., 2022). Furthermore, current interest in environmental aspects have also been enhanced by using MALDI-MSI to add further details. For example, in salinity stress large changes were found in the PC profiles of salt-treated roots. These data were suggested to provide a promising way in which plants could combat abiotic stresses such as those of increased salinity (Sarabia et al., 2018).

## 7 Conclusions and future perspectives

It is clear that research into lipid production in *B. napus* and closely related *Brassica* oil crops is very active. While the overall pathway for triacylglycerol biosynthesis and oil accumulation is known and relevant enzymes have been studied, specific details about genetic regulation are continuing to be uncovered. Indeed, it is in the area of transcriptional regulation that much of the recent work has concentrated. Many of the latest publications have used Arabidopsis as a surrogate plant for *B. napus* but, as pointed out before, the comparison has to be made with caution (Li et al., 2006). Nevertheless, when discoveries in Arabidopsis are transferred to *B. napus*, the results often prove useful in terms of altering oil accumulation and, sometimes, its composition. Because agricultural land is limiting (and may well be shrinking due to climate change) while global populations increase, efforts to increase productivity of existing crops is extremely important. *B. napus* as a key oil crop in temperate climates is vital in this regard.

Some aspects summarized in this review have had important recent advancements and merit further study. In particular, the use of flux control studies can provide relevant information to pinpoint useful parts of metabolism which can be manipulated to increase oil accumulation (Fell et al., 2023; Weselake et al., 2024). Environmental influences on crop yields in *B. napus* have only recently been studied in much detail and, with the acute and chronic effects of climate change becoming all too obvious, they deserve more work. Furthermore, new and/or improved techniques can reveal novel aspects of biochemistry which can have important temporal or spatial significance. This is exemplified by the MALDI-MS work illustrated in this review. Further advances in our knowledge of *B. napus*, as a vital oil crop, are confidently expected in the future.
